# Carbolic Acid in Vascular Keratitis

**Published:** 1871-06

**Authors:** C. Hixon

**Affiliations:** Formerly Professor of Opthalmology in the College of Physicians and Surgeons, Kansas City, Mo.


### CARBOLIC ACID IN VASCULAR KERATITIS.

By C. HIXON, M.D., formerly Professor of Ophthalmology in the College of Physicians and Surgeons, Kansas City, Mo.

In medicine, theories are of little value compared with well-attested facts. No one can, from a process of reasoning, say that any given agent will do so and so; consequently the remedial value of any given agent, simple or compound, must be discovered, if discovered at all, by the test of actual experimentation. These, among other considerations, led me to use carbolic acid in the treatment of the following case. I know of no case on record where this agent has been used in the treatment of this disease, and it is as much to induce others to give it a trial as anything else that prompts me to report the case:—

Mrs. G., aged 23, of delicate frame and rheumatic diathesis, came to consult me on the 1st of February last. She lives about twenty miles from this city, and has been under the care of a general practitioner who pays but little attention to the eye, and under whose treatment she was severely ptyalised. This mercurial action, together with a lactation of fifteen months' duration, had rendered her extremely exsanguinous and emaciated.

On examination I found the following: Moderate thickening of the palpebral conjunctiva, the ocular conjunctiva much more extensively involved—extreme vascular keratitis (the iris entirely hidden)—a large ulcer on the lower and outer half of the cornea, extending deep into the corneal luminæ—great photophobia, it being impossible for the patient to open the eye herself—profuse lachrymation, with periods of great suffering from circumorbital neuralgia. The enlarged episcleral vessels and circumcorneal zone indicated iritis, with perhaps inflammation of other intraocular tissues, but the dense corneal opacity rendered an accurate diagnosis upon this point impossible, and could only be arrived at by rational symptoms.

Owing to her prostrated condition little else was attempted for four weeks than to repair the general health by tonics and good living, and at once removing the child from the breast. At the expiration of this time her general condition had materially improved, but no perceptible improvement in the eye except as to the pain, which, she stated, seemed not so severe. I now applied to the corneal ulcer a couple of drops of the solution of carbolic acid in glycerine, of the strength of sixty grains to the ounce of glycerine. This I had frequently used before in ulcers of the cornea that were indolent and very painful, and with the very best effect. I believe this practice originated with Dr. Williams, of Cincinnati. The immediate effect of the application was excruciating pain for a moment, and a complete whitening of the whole cavity of the ulcer from the coagulation of albumen. The day following I did the same, and continued to do so for three weeks, by which time the ulcer had entirely healed, when the acid was more sparingly applied. The pain on its application became less and less as the ulcer disappeared and the cornea cleared up. So soon as the cornea became sufficiently transparent lateral illumination revealed partial occlusion of the pupil, and mydriatics discovered synechia posterior, which in time yielded to atropia, except at one point, which has so far resisted all efforts. The vascularity of the cornea has entirely disappeared, but, of course, a leucoma remains in the site of the ulcer.

The general treatment consisted of iron, nitric acid, and quinia, and to meet the rheumatic element in the case the following was used:

℞. Vin. Colch. sem. ........................ } āā f℥ijss.

Ext. Phytolac, fl. ........................ }

Tr. Stramonium ........................ f℥i.

Tr. Aconit, fol. ........................ f℥ss.

Sig. A teaspoonful three times a day. Under this treatment she made a rapid recovery. I report this case, believing it to be the first case where carbolic acid has been used in the treatment of vascular keratitis. If others have used it I am not aware of it, and should be happy to hear. I certainly shall continue to test its powers in similar cases.

